# Supplementary material for: Antipsychotic Chlorpromazine Suppresses STAT5 Signaling, Overcomes Resistance Mediated by the Gatekeeper Mutation FLT3-ITD/F691L, and Synergizes with Quizartinib in FLT3-ITD-Positive Cells
Source: Curr Issues Mol Biol. 2025 Sep 25;47(10):797. doi: 10.3390/cimb47100797 (PMC12564577; doi:10.3390/cimb47100797)
Supplement: Supplementary file 1 [file cimb-47-00797-s001.zip › cimb-3850289-supplementary.pdf]

Supplementary Figure S1

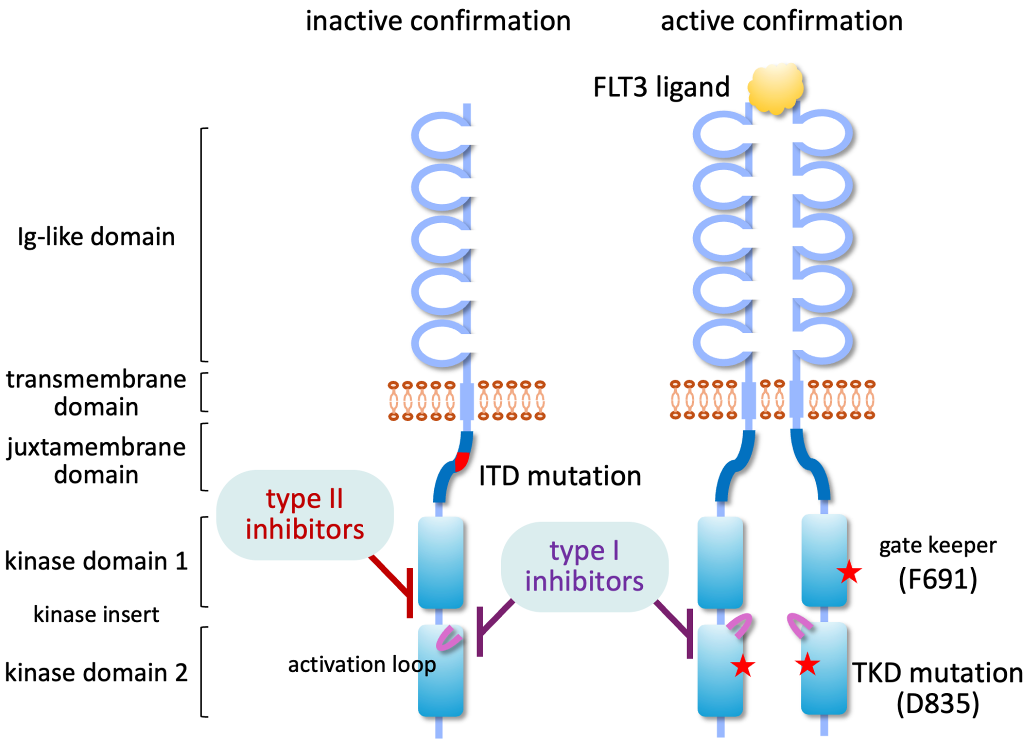


**Domain architecture of FLT3, highlights common ITD and TKD, and depicts representative inhibitor binding modes**

FLT3 consists of five extracellular Ig-like domains, a juxtamembrane domain (JMD), two tyrosine kinase domains (TKDs), and a C-terminal region. Ligand (FL) binding induces dimerization, TKD phosphorylation, and downstream signaling essential for hematopoietic cell differentiation, proliferation, and stem cell self-renewal. In leukemic cells, FL stimulation promotes proliferation and suppresses apoptosis. Activating mutations include ITD in the JMD (20–28%) and TKD mutations such as D835 (5–10%), both leading to constitutive signaling. FLT3 inhibitors competitively bind to the ATP-binding site, thereby blocking kinase activity and inducing apoptosis. However, the conformation of the ATP-binding pocket differs between inactive and active states. Type I inhibitors bind either the ATP-binding site of constitutively active receptor (via ITD or TKD mutations) or the activation loop located between the TKDs, thereby blocking signaling. Type II inhibitors bind to a region adjacent to the ATP-binding site in the inactive kinase conformation, thereby inhibiting phosphorylation driven by ITD mutations. The F691L gatekeeper mutation arises in TKD1 and confers resistance to many FLT3 inhibitors, making it a critical therapeutic challenge.
